# Surface Interactions between Ketoprofen and Silica-Based Biomaterials as Drug Delivery System Synthesized via Sol–Gel: A Molecular Dynamics Study

**DOI:** 10.3390/ma15082759

**Published:** 2022-04-08

**Authors:** Giuseppina Raffaini, Michelina Catauro

**Affiliations:** 1 Department of Chemistry, Materials, and Chemical Engineering ‘‘Giulio Natta’’, Politecnico di Milano, Piazza L. Da Vinci 32, 20131 Milano, Italy; 2 Department of Engineering, University of Campania “Luigi Vanvitelli”, Via Roma 29, 81031 Aversa, Italy

**Keywords:** biomaterials, silica-based materials, surface interactions, molecular dynamics simulations, adsorption, drug loading, drug delivery, ketoprofen

## Abstract

Biomaterial-based drug delivery systems for a controlled drug release are drawing increasing attention thanks to their possible pharmaceutical and biomedical applications. It is important to control the local administration of drugs, especially when the drug exhibits problems diffusing across biological barriers. Thus, in an appropriate concentration, it would be released in situ, reducing side effects due to interactions with the biological environment after implantation. A theoretical study based on Molecular Mechanics and Molecular Dynamics methods is performed to investigate possible surface interactions between the amorphous SiO_2_ surface and the ketoprofen molecules, an anti-inflammatory drug, considering the role of drug concentration. These theoretical results are compared with experimental data obtained by analyzing, through Fourier transform infrared spectroscopy (FT-IR), the interaction between the SiO_2_ amorphous surface and two percentages of the ketoprofen drug entrapped in a silica matrix obtained via the sol–gel method and dried materials. The loaded drug in these amorphous bioactive material forms hydrogen bonds with the silica surface, as found in this theoretical study. The surface interactions are essential to have a new generation of biomaterials not only important for biocompatibility, with specific structural and functional properties, but also able to incorporate anti-inflammatory agents for release into the human body.

## 1. Introduction

In the field of biomaterials, a great interest is covered by nanoparticle-based drug delivery systems for treating different diseases [1,2,3,4,5]. These nanoparticles may be biocompatible, stable, and biodegradable, and they should not be cytotoxic or, in general, provoke an immune response [6]. Inorganic materials often used to form nanoparticle systems include iron, gold, silica, silver, titanium, and zinc [7,8]. Mesoporous silica nanoparticles (MSNs) have recently received a lot of interest because of their drug carrier systems, bio-medicine potential as targeting systems, bio-imaging, and controlled drug release [9,10,11]. It was found that the adsorption capacity of sorbents with high silica content is related to their surface hydrophobicity (hydrophilicity) and structural features, such as micro-pore volume and pore size. Over the last few years, a rapid increase in research on MSNs as drug carriers for the treatment of various diseases has been observed, indicating its potential benefits in drug delivery. When using porous silica biomaterials for drug release, the drug is continuously released to maintain the drug concentration required for effective treatment in order to have a long and sustained drug release time for disease treatment, which can avoid the side effects caused by the traditional drug delivery system [12].

The sol–gel method is an interesting technique useful to prepare micro- or nanoporous silica-based materials with different percentages of drugs, and thanks to spectroscopic techniques, it is possible to have useful information about the surface interactions with drug molecules [13,14,15,16]. The synthesized silica nanoparticles can be used as coating additives, and they can modify the surface properties of organic, usually polymeric coatings. Interestingly, silica nanoparticles increase the water uptake at the polymeric interface, and this fact is very important for drug release and full cell applications, enhancing the desirable drug release or proton transfer [17,18,19]. Moreover, thanks to their possible pharmaceutical and biomedical applications, silica-based systems can control the local drug delivery becoming systems for anti-inflammatory agents. In fact, these systems have extended therapeutic effects and reduced side effects, controlling the release during the time and the amount of drug on the biomaterials surfaces in situ.

Mesoporous silica nanoparticles proved to be a very promising drug delivery carrier that can be used as a facile way to enhance the bioavailability of poorly soluble drugs such as ketoprofen solubilizing dispersing it then in an amorphous silica nanostructure thanks to favorable non-covalent interactions at the external surface of porous material [20]. Silica–Alginate beads, for example, are studied for intestinal ketoprofen delivery [21]. Magnetic core-mesoporous silica nanoparticles were prepared and coated with a dense silica layer in order to encapsulate ketoprofen drugs [22], and mesoporous silica material was synthesized using different ratios of ketoprofen and characterized as sorbents for matrix solid-phase dispersion (MSPD) systems [23]. Interestingly, plain silica gel layers with an achiral mobile phase are studied as a useful stationary phase for the enantioseparation of *S*,*R* (±)-ketoprofen, confirming interesting intermolecular interactions between ketoprofen molecules and silica surface [24], interactions that can be theoretically modeled.

Given the great attention to the study of intermolecular surface interactions between nanoparticles and drug molecules, in this work, using theoretical methods based on Molecular Mechanics (MM) and Molecular Dynamics (MD) simulations at the atomistic level, the study of the interactions between biomaterial silica surface and drug molecules is reported. Usually, in studies, a lot of papers are reported related to the conformational properties of drug molecules, their interaction with systems able to solubilize them [25,26,27], for example, hydrophobic anticancer drug molecules or anti-inflammatory drugs using β-cyclodextrins (CDs) [28,29], and CD complexes that self-associate to form an aggregate or micelle-like structures [30,31] or β-cyclodextrins nanosponges [32,33,34]. In the biomaterial literature papers, it is considered the interaction between proteins or their fragments and biomaterial surfaces in order to understand the biocompatibility related to conformational changes in the tertiary structure of the adsorbed protein, in the blood or in contact with bone tissue, for example, comparing experimental data and theoretical data [35,36,37,38,39,40,41,42,43,44,45,46].

A recent interesting theoretical paper deals with the insulin adsorption on crystalline and amorphous SiO_2_ using accelerated dynamics simulations proposed by Nejad et al. [47]. In this work, it is highlighted that considering apolar surfaces, the van der Waals contribution is the driving force of the adsorption process [36], while on polar surfaces such as crystalline or amorphous SiO_2,_ the electrostatic contribution plays a key role in the potential energy, favoring the adhesion at the interface, as also found in this theoretical work.

Bulk properties and surface properties, order or disorder, and wettability can play an important role in the adsorption process, and they must also be better investigated theoretically in order to better highlight the key factors that affect the adsorption and/or reversible desorption process, or diffusion [48,49]. Atomistic simulations can be a useful tool to well describe the surface chemistry and, in particular, its nano-topography and the interaction with a specific drug molecule, also considering the racemic mixture of carriers of drugs on chiral DNA double-stranded architecture. Drug nanocarriers might increase the solubility of hydrophobic drugs in aqueous solution by loading drug molecules into pores or hydrophobic layers of nanocarriers [12]. The interaction between SiO_2_ surfaces due to drug molecules using atomistic modeling based on MM and MD simulations is absent in studies and interesting in this work is the useful comparison with FT-IR experiments to identify the nature of the intermolecular interactions on the silica surface. Moreover, there are no theoretical studies at the atomistic level considering also the importance of the concentration, focusing the attention on the possible different drugs dispersion on the biomaterial surface, an important factor for good release during the time in situ, with good dispersion of drugs or at a higher concentration as self-organized nanoaggregates. Intermolecular interactions, depending on the drug concentration, can play an important role both for the release during the time depending on the entity of the interaction and also for the cytotoxicity when in situ the amounts of the drug are too large. Using a simulation protocol proposed in previous work about protein adsorption on biomaterial surface [36,37,38,39,40,41] or about cyclodextrin or CD nanosponge model for drug release [30,31,33] interacting with the hydrophobic drugs both in hydrophobic β-CD cavity and on the nanosponge surface, in these work the adsorption of ketoprofen molecule on SiO_2_ amorphous surface is studied and compared with FT-IR experimental data, confirming the presence of drug well dispersed on the solid surface forming H-bonds drug-biomaterial for drug release.

Then, in this work, a theoretical study based on Molecular Mechanics and Molecular Dynamics methods is performed to investigate the intermolecular interactions between an amorphous SiO_2_ surface and the ketoprofen drug molecules, a non-steroidal anti-inflammatory drug (NSAID), both at small and higher concentration to evaluate both the surface interaction and the role of drug concentration, by comparing the theoretical results with experimental data. In fact, a system composed of SiO_2_ glass and ketoprofen was synthesized via sol–gel process considering two percentages of the drug (5 and 15 wt. %) entrapped and dried materials were analyzed through Fourier transformed infrared spectroscopy (FT-IR). The drug-loaded amorphous bioactive materials form hydrogen bonds with the silica surface, as found in this theoretical study, and some H-bonds among drug molecules at larger concentrations, but are always well dispersed on silica-based material. This surface interaction is important in order to have a new generation of biomaterials not only important for structural and functional properties but also for the ability to incorporate anti-inflammatory agents for release into the human body.

## 2. Materials and Methods

### 2.1. Materials

Tetraethyl orthosilicate reagent grade (TEOS, (C_2_H_5_O)_4_Si) and Ethanol 99.8% (EtOH), provided by Sigma-Aldrich (Darmstadt, Germany), were used for the Sol–Gel synthesis within MilliQ water and ketoprofen 98.0%, provided by ChemPUR (Karlsruhe, Germany). Potassium Bromide 99.0% (KBr), provided by Sigma-Aldrich (Darmstadt, Germany), was used for FT-IR analyses.

### 2.2. Methods

#### 2.2.1. Silica/Ketoprofen (5 and 15 wt. %) Sol–Gel Synthesis

The Sol–Gel procedure, used to obtain the hybrid silica/ketoprofen (5 and 15 wt. %), could be divided into five steps: (i) preparation of silica solutions (Sols A); (ii) preparation of ketoprofen solutions (Sols B and C); (iii) mixing Sols A with B or C; (iv) gelation and (v) drying times. Firstly, TEOS was dissolved into EtOH and MilliQ H_2_O (Sols A) under continuous stirring, whereas ketoprofen solutions, 5 wt. % (for Sol B) and 15 wt. % (for Sol C) with respect to silica content, were prepared by mixing ketoprofen with EtOH. After the complete Sols formations, Sols B and C were added drop by drop to Sols A under continuous magnetic stirring. The molar ratios of solutions were H_2_O/TEOS = 0.9 and EtOH/TEOS = 2.8 for SiO_2_/ketoprofen 5 wt. %, while H_2_O/TEOS = 0.9 and EtOH/TEOS = 5.5 for SiO_2_/ketoprofen 15 wt. %. After the gelation occurrences, the gel was dried at 50 °C for 24 h, obtaining the hybrid biomaterials.

#### 2.2.2. Fourier Transform Infrared Analysis

FT-IR analysis was performed in the range of 400–4000 cm^−1^ using the Prestige21 Shimadzu system, equipped with a DTGS KBr (deuterated triglycine sulfate with potassium bromide windows) detector, resolution of 2 cm^−1^ (60 scans). The analysis procedure uses KBr disks (2 mg of sample and 198 mg of KBr).

#### 2.2.3. Molecular Mechanics and Dynamics Methods

The theoretical study is based on Molecular Mechanics (MM) and Molecular Dynamics (MD) methods adopting the same simulation protocol proposed in previous work [36,37,38,39,40,41]. All simulations were carried out using the Materials Studio package (Accelrys Inc., San Diego, CA, USA) and the CVFF force field [50,51,52]. At atomistic level, at first, the (*R*,*S*) ketoprofen enantiomer molecules, then the amorphous SiO_2_ thin film using the amorphous structure reported in Materials Studio structures were modeled and considered fixed in order to study the nature of the surface interactions with the chosen drug. In the second step, the adsorption process of ketoprofen drug molecules on the hydrated biomaterial surface, both at small and at larger drug concentrations, was studied. In the third step, in order to mimic the experimental procedure, starting from stable hydrated surface with drug molecules in contact with silica surface, the water molecules are deleted, and another energy minimization, MD run, and geometry optimization of the final geometry at equilibrium achieved in vacuo were started, thus considering the dried surface, as performed in the experimental procedure before FT-IR experiments. All simulations are carried out using periodic boundary conditions in the NVT ensemble, considering at constant temperature equal to 300 K controlled through the Berendsen thermostat in a simulation box of size equal to (85.53 × 85.53 × 250.0) Å. All energy minimizations were carried out using the *Conjugate Gradient* algorithm up to an energy gradient lower than 4 × 10^−3^ kJ mol^−1^ Å^−1^. Using a simulation protocol proposed in previous work, after an initial energy minimization, MD runs lasting 2 ns, and final geometry optimizations and the adsorption at small and at larger concentration was studied, both considering hydrated surface and finally the dried surface. The integration of the dynamical equations was carried out with the Verlet algorithm using a time step of 1 fs.

After the conformational study of (*R*,*S*) ketoprofen enantiomers, the results of the adsorption process of molecules at small and at higher concertation on the amorphous SiO_2_ biomaterial surface were compared with experimental data from IR spectra obtained on SiO_2_ synthesized by sol–gel methods. The study of the surface interactions between ketoprofen molecules and the amorphous SiO_2_ surface indicates the formation of H-bonds between the drug and the surface. In particular, at small concentrations, a racemic mixture of 14 (*R*,*S*) ketoprofen molecules was considered, and 28 (*R*,*S*) ketoprofen molecules were at higher concentrations. In the next section, the results will be presented and discussed. Considering the silica surface area exposed to the solvent and the surface area exposed by all the drug molecules chosen, 14 and 28 ketoprofen molecules allow to avoid the total surface coverage and also consider the possible hydrophobic interactions between the aromatic rings in drug molecules when in greater number without total surface coverage as in the experimental study.

## 3. Results and Discussion

**Figure 1:**
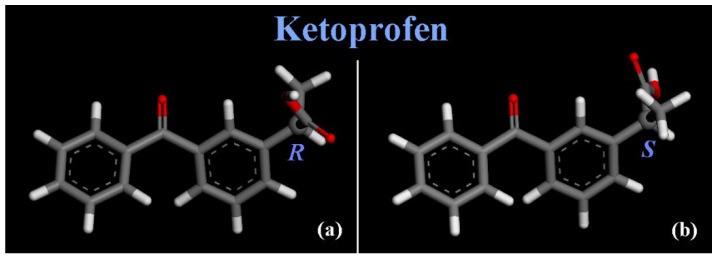
Optimized geometries of ketoprofen drug molecules, in particular the (*R*) enantiomer in panel (**a**) and the (*S*) enantiomer (*S*) in panel (**b**) are shown. Color code: carbon atoms are in grey, oxygens in red and hydrogen atoms in white.

The results of the adsorption process of (*R*,*S*) ketoprofen racemic mixture on amorphous SiO_2_ surface will be discussed in Section 3.1 considering ketoprofen at small concentration, in Section 3.2 considering ketoprofen at higher concentration, and then the comparison with FTIR spectra in Section 3.3. The stable conformations of (*R*,*S*) ketoprofen drug molecule optimized geometries are reported in Figure 1.

### 3.1. (R,S) Ketoprofen Molecules on Amorphous SiO_2_ Surface: Small Drug Concentration

Reproducing in the theoretical work the same experimental procedure used for FTIR measurements, the results obtained after MM and MD simulations about the adsorption process of (*R*,*S*) ketoprofen racemic mixture on amorphous SiO_2_ at small concertation will be discussed in Section 3.1 considering the hydrated surface, then considering the dried surface in vacuo, mimicking the same experimental procedure for FTIR data.

**Figure 2:**
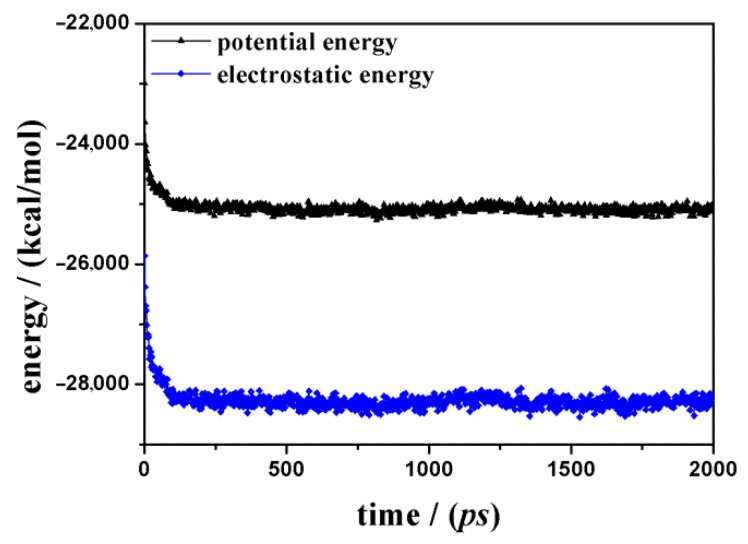
Potential energy (line and symbols in black) and electrostatic energy (line and symbols in blue) calculated as a function of the MD run time starting from 14 ketoprofen drug molecules in racemic mixture on hydrated amorphous SiO_2_ surface are shown.

At first, the adsorption of the racemic mixture containing 14 ketoprofen drug molecules considering a layer of water molecules 15 Å thick near the SiO_2_ surface was investigated. When using a simulation protocol proposed in previous work [36,37,38,39,40,41], after energy minimization, an MD run lasting 2 ns, a layer of water molecules containing drug molecules adsorbed on an amorphous SiO_2_ surface is characterized. The potential energy calculated during the MD run is reported in Figure 2; in particular, the fast decrease in the potential energy is due to the important contribution of the electrostatic energy, as found by Nejad et al. [47] about the adsorption of insulin on polar SiO_2_ silica surface.

**Figure 3:**
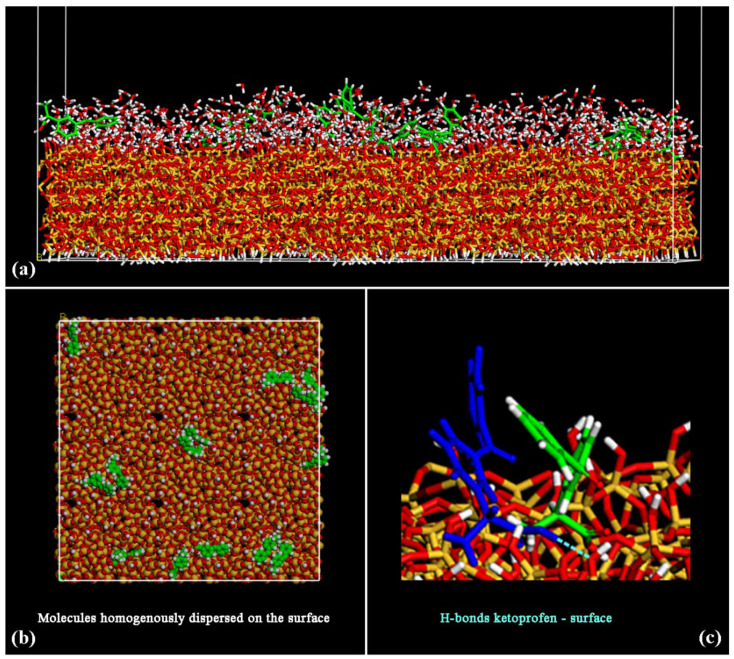
Optimized geometry after MD run of 14 ketoprofen drug molecules in racemic mixture adsorbed on hydrated amorphous SiO_2_ surface, in particular, the side view is in panel (**a**) and the top view in panel (**b**) without water molecules for clarity. Color code: carbon atoms are in green, oxygens in red and hydrogen atoms in white. In panel (**c**) the atoms of ketoprofen molecule that form an H-bond with the SiO_2_ surface are reported in blue.

During the MD simulations, the water and the drug molecules come close to the surface and interact together, forming H-bonds. In Figure 3, the side view of the final optimized geometry after MD run and energy minimization of the system at the end of dynamics simulations at room temperature is reported in panel (a), the top view in panel (b). In panel (c), a particular about the formation of H-bonds between a ketoprofen molecule and the hydrated silica surface take place (see dashed light blue line between blue ketoprofen molecule and an oxygen atom of SiO_2_ surface of Figure 3, without water molecules for clarity).

After the MD run, the adsorbed ketoprofen and the water molecules interact with the surface. The hydrophilic SiO_2_ surface interacts with the water molecules that form a hydration layer. At the end of the MD run, the drug molecules are well dispersed on the surface, as reported in the top view in panel (b) of Figure 3. The ketoprofen molecules interact with the surface forming some H-bonds as reported in panel (c). Only two pairs of ketoprofen drugs interact together thanks to hydrophobic interactions between their aromatic rings.

**Figure 4:**
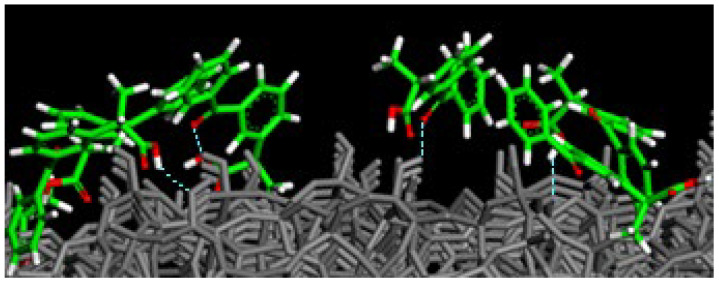
Particular of the optimized geometry of 14 ketoprofen drug molecules in racemic mixture adsorbed on dried amorphous SiO_2_ surface, then without water molecules, after 2 ns in vacuo and energy minimization. In particular the Figure shows the side view of ketoprofen molecules interacting with SiO_2_ surface forming H-bonds. Color code: all surface atoms are in grey, ketoprofen carbon atoms are in green, oxygens in red and hydrogen atoms in white. H-bond with the SiO_2_ surface is reported in dashed blue lines.

After the study of the adsorption process on the hydrated amorphous SiO_2_ surface, the water molecules are deleted, and the interaction on the dried surface using the same theoretical procedure is now investigated. After energy minimization, an MD run lasting 2 ns and energy minimization of the final geometry assumed by the system at the end of the MD run in vacuo, mimicking the dried silica surface after the drug loading, the ketoprofen molecules are closer to the surface forming H-bonds both between C=O_carboxylic_⋯H-O_silica surface_ and O-H_carboxylic acid_⋯O-H_silica surface_, as reported in Figure 4. Drug molecules are again homogeneously dispersed on the silica surface. Only two pairs of ketoprofen drugs interact thanks to hydrophobic interactions between their aromatic rings.

### 3.2. (R,S) Ketoprofen Molecules on Amorphous SiO_2_ Surface: Higher Drug Concentration

The results of the adsorption process of (*R*,*S*) ketoprofen racemic mixture on amorphous SiO_2_ at higher concertation will be discussed in Section 3.2 at first considering a hydrated surface, then the dried surface after MM and MD simulation in vacuo.

**Figure 5:**
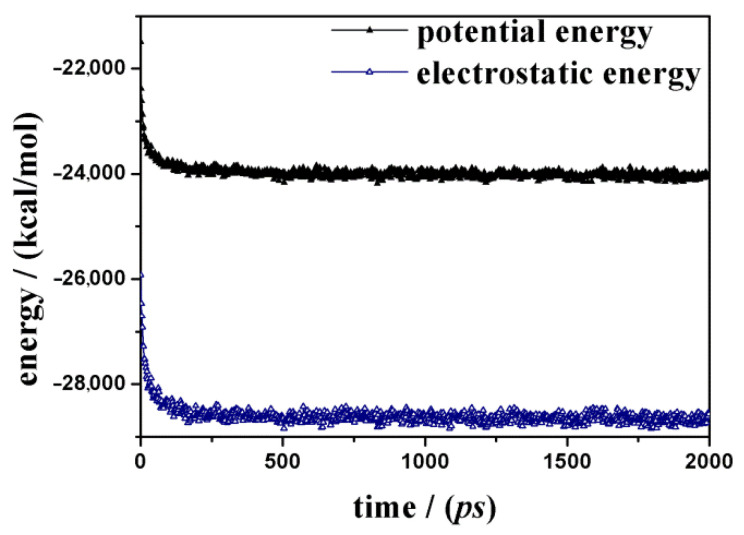
Potential energy (line and symbols in black) and electrostatic energy (line and symbols in blue) calculated as a function of the MD run time starting from 28 ketoprofen drug molecules in racemic mixture on hydrated amorphous SiO_2_ surface are shown.

When using the same simulation protocol used in the previous section, at first, the adsorption of a racemic mixture containing 28 ketoprofen drug molecules dispersed in a 15 Å thick layer of water molecules near the amorphous SiO_2_ surface was investigated. Figure 5 shows the potential energy values calculated during the MD run and the electrostatic energy, pointing again to the importance of this contribution.

**Figure 6:**
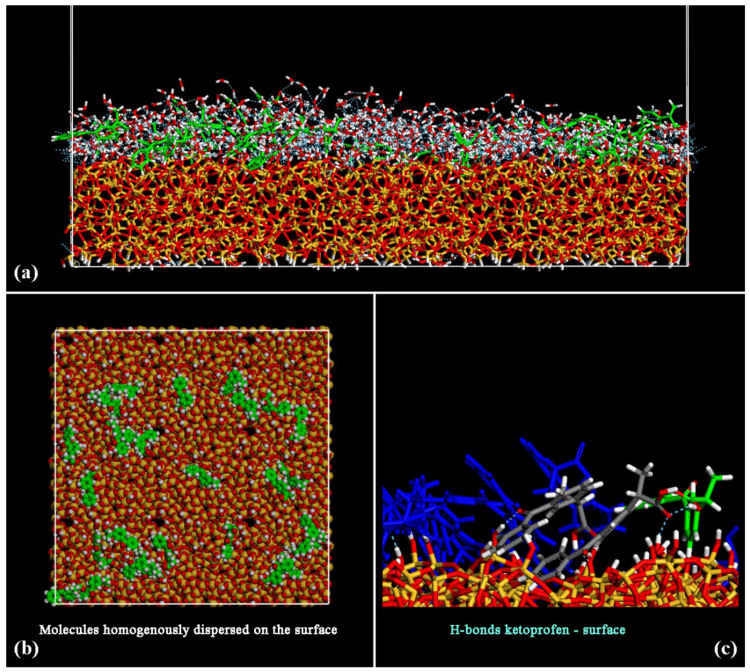
Optimized geometry after MD run of 28 ketoprofen drug molecules in racemic mixture adsorbed on hydrated amorphous SiO_2_ surface, in particular side view in panel (**a**) and topo view in panel (**b**) without water molecules for clarity. For panels (**a**) and (**b**) the color code is the same of Figure 3. In panel (**c**) some ketoprofen molecules are in blue, some with carbon atoms in grey or in green in order to better show possible H-bonds in dashed light blue lines.

After energy minimization and an MD run lasting 2 ns with a layer of water molecules adsorbed on an amorphous SiO_2_ surface, the drug molecules interact with the silica surface (see panel (a) in Figure 6). In this case, at larger concentrations, two or more ketoprofen molecules interact together, thanks to hydrophobic interactions, but, in general, are again homogeneously dispersed on the silica surface (see the top view in panel (b) in Figure 6), forming H-bonds both with the surface, with water molecules and with drug molecules (see dashed light blue lines in panels (a) and (c) in Figure 6).

**Figure 7:**
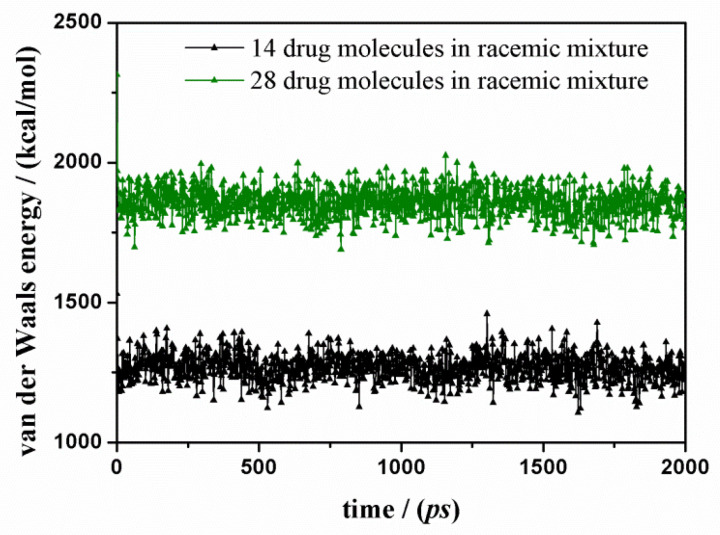
Van der Waals energy contributions calculated as a function of the MD run time starting from 14 and 28 ketoprofen drug molecules in racemic mixture on hydrated amorphous SiO_2_ surface respectively green and black line and symbols.

To better understand the driving force of the adsorption process on this polar surface, in Figure 7, the van der Waals contributions calculated during MD simulations in water both at small and at higher concentrations indicate that hydrophobic interactions take place but fluctuate around the average value during the MD run. The hydrophobic interactions take place when considering the ketoprofen molecules, which are well dispersed on the polar surface but can interact when aromatic rings are at shorter distances. Interestingly, the main driving force of the adsorption process on the polar amorphous SiO_2_ surface is then once more the electrostatic forces, as reported in Figure 2 and Figure 5. In these two graphs, the trend is the same: a similar initial fast decrease and then fluctuation around an average value. Figure 2 and Figure 5 show the fundamental contribution to potential energy due to electrostatic energy that is the driving force of the adsorption process on this polar surface, as reported by Nejad et al. [47] about the adsorption on crystalline and amorphous SiO_2_ surfaces. Electrostatic interactions are due to the alignment of ketoprofen dipoles with surface dipoles, forming H-bonds that during MD run are continuously formed and disrupted due to kinetic energy at 300 K, always maximizing on the surface these interactions. The different values of specific electrostatic and potential energies are due to the different number of drug molecules in a simulation box.

**Figure 8:**
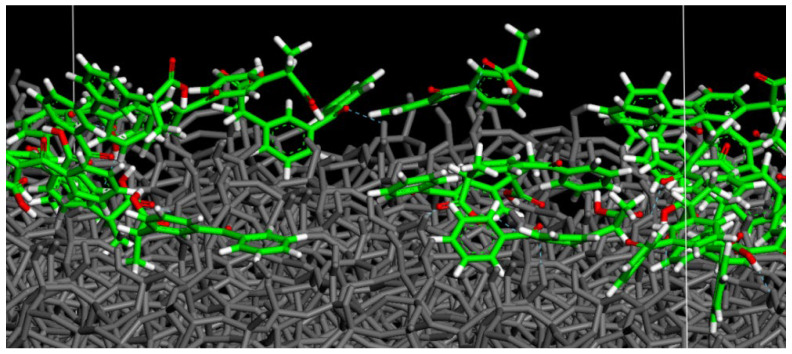
Particular of the optimized geometry of 28 ketoprofen drug molecules in a racemic mixture adsorbed on a dry amorphous SiO_2_ surface, without water molecules, after 2 ns in vacuo and energy minimization. In particular the Figure shows the side view of ketoprofen molecules interacting with SiO_2_ surface forming H-bonds. Color code: all surface atoms are in grey, ketoprofen carbon atoms are in green, oxygens in red and hydrogen atoms in white. H-bond with the SiO_2_ surface is reported in dashed blue lines.

After the study of the adsorption process on hydrated SiO_2_ surface, again, using the same procedure at a small drug concentration, the water molecules are deleted, and afterward, the interaction on the dried silica surface loaded by drug molecules is studied. After an MD run lasting 2 ns and energy minimization in vacuo ketoprofen molecules without water on the amorphous silica surface, it was found that the drug molecules are closer to the dried amorphous SiO_2_ surface forming H-bonds both between C=O_carboxylic_⋯H-O_silica surface_ and O-H_carboxylic acid_⋯O-H_silica surface_ as reported in Figure 8. Moreover, the drug molecules have again a homogeneous surface distribution similar to that found in the presence of water molecules but closer to the dry surface.

### 3.3. FTIR Spectra

**Figure 9:**
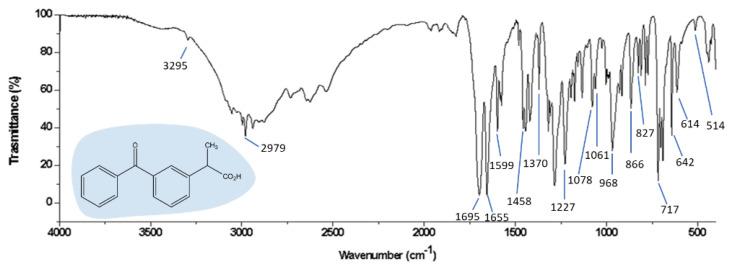
FT−IR spectrum of Ketoprofen.

**Figure 10:**
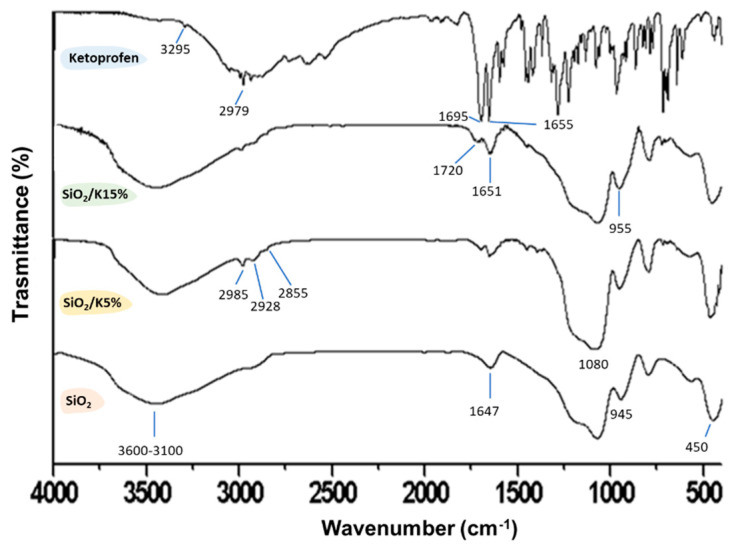
FT−IR spectra of Ketoprofen, SiO_2_/Ketoprofen (5 and 15 wt. %) hybrid materials and pure SiO_2_.

Two drug percentages (5 and 15 wt. %) were entrapped in a silica matrix synthesized via the sol–gel method. After the drying procedure, the obtained materials were analyzed through FT-IR. FT-IR analysis was performed in the range of 400–4000 cm^−1^ using the Prestige21 Shimadzu system, equipped with a DTGS KBr (deuterated triglycine sulfate with potassium bromide windows) detector, resolution of 2 cm^−1^ (60 scans). The analysis procedure uses KBr disks (2 mg of sample and 198 mg of KBr). FT-IR spectra were reported in Figure 9 and Figure 10, respectively. In particular, Figure 9 reports the FT-IR spectrum of ketoprofen. According to Vueba et al. [53], whom in-depth described both the signals of this drug by distinguishing the bands that belonged from the two (*S*,*R*) ketoprofen enantiomers, the peak at 3295 cm^−1^ is assigned to O-H stretching, the peak at 2979 cm^−1^ is assigned to C-H stretching, while the peaks at 1695 and 1655 cm^−1^ are assigned to C-O stretching modes.

FT-IR spectra of ketoprofen, SiO_2_/ketoprofen (5 and 15 wt. %), and pure SiO_2_ are reported in Figure 10. After the syntheses of silica/ketoprofen hybrid materials via sol–gel, it is possible to notice that some FT-IR ketoprofen peaks disappear after the synthesis, suggesting the occurrence of the drug entrapment. The formation of the hybrid is also confirmed by the co-presence of SiO_2_ characteristic peaks (Si-O-Si asymmetric stretching at 1080 cm^−1^, Si-OH stretching, and -OH stretching at 3400–3100 cm^−1^, Si-O bending at 450 cm^−1^ [54,55,56,57]) and some ketoprofen slightly shifted peaks. Indeed, the peaks at 2979 cm^−1^ and 1697 shift slightly to higher wavenumbers (2985 cm^−1^ and 1720 cm^−1^), while the signal at 1655 cm^−1^ shifts to lower wavenumber (1651 cm^−1^). When comparing the hybrid spectra with the ones of ketoprofen and silica alone, it can be observed that there are changes in the intensity and shape of signals in the range of 3600–3100 cm^−1^ and at 1651 cm^−1^. These changes can be attributed to the formation of H-bonds between the C-O and -OH ketoprofen groups with the hydroxyl groups placed on the silica matrix surface, supporting as demonstrated by MM and MD models.

## 4. Conclusions

MM and MD atomistic simulations are performed in order to investigate surface interactions between the amorphous SiO_2_ surface and the (*R*,*S*) ketoprofen molecules, an anti-inflammatory drug, both at small and at higher concentration, at first on the hydrated amorphous silica surface, then on the dried surface loaded by drug molecules, as performed in the experimental procedure. In fact, at first, the adsorption process was simulated on a hydrated amorphous SiO_2_ surface, then on dried silica material, mimicking the same procedure experimentally used before the FTIR analysis of two percentages of ketoprofen drug entrapped in a silica matrix obtained via the sol–gel method and dried materials. The drug that is loaded in these amorphous bioactive materials forms hydrogen bonds with the silica surface as indicated in the FT-IR spectra and as calculated by the theoretical study. The drug molecules are homogeneously dispersed on the amorphous silica surface, and, at higher concentrations, some hydrophobic interactions between drugs molecules are found, being otherwise well dispersed on the silica surface. These surface interactions are important in order to have a new generation of biomaterials that are not only important for biocompatibility, with specific structural and functional properties [48], but also able to incorporate anti-inflammatory agents for release in situ into the human body, thanks to weak intermolecular interaction, such as the H-bonds, among the polar SiO_2_ biomaterials surface and anti-inflammatory agents. The intermolecular interactions play a key role, and on this basis, it is possible to improve the synthesis and the applications aimed at obtaining the best drug loading, release, bioactivity, and avoiding adverse side effects.

Biomaterials as drug delivery systems are potentially important either to reduce the inflammatory responses in situ in a biological environment and the cytotoxicity, control the drug that is loaded and its availability for release over time, overcoming the problem of inhibition or very slow drug diffusion through biological membranes. Ketoprofen is potentially released from silica in situ thank to non-covalent interactions with proteins in a biological environment. In fact, the first crucial step of biomaterials in contact with the biological systems is the adsorption on the external biomaterial surface.

These novel systems based on amorphous silica surfaces loaded with drug molecules can have an interesting impact in the field of disease therapy as all hybrid porous nanomaterials, and the atomistic modeling and dynamics simulations at the interface, provide new insights about biomaterial nanocarriers for drug release.

## Abbreviations

MSN	Mesoporous Silica Nanoparticle
MM	Molecular Mechanics
NSAID	Nonsteroidal anti-inflammatory drug
MSPD	Matrix solid-phase dispersion
MD	Molecular Dynamics
H-bond	Hydrogen bond
NVT ensemble	Number of particles, Volume and Temperature are constant
